# Impact of sample mass on the reliability of toenail fluoride for exposure assessment

**DOI:** 10.1007/s13530-026-00321-y

**Published:** 2026-04-27

**Authors:** Guillermo Tamayo-Cabeza, Michael Zenni, Gina A. Castiblanco-Rubio, Frank Lippert, David B. Flora, Janet L. Peacock, Christine Till, Carly V. Goodman, Bruce P. Lanphear, Susan A. Korrick, Margaret R. Karagas, E. Angeles Martinez-Mier

**Affiliations:** 1 Department of Dental Public Health and Dental Informatics, School of Dentistry, Indiana University Indianapolis, Indianapolis, IN USA; 2 Department of Sociology, College of Liberal Arts, Purdue University, Indianapolis, IN USA; 3 Department of Biomedical and Applied Sciences, Oral Health Research Institute, School of Dentistry, Indiana University Indianapolis, Indianapolis, IN USA; 4 Department of Psychology, York University, Toronto, ON Canada; 5 Department of Epidemiology, Geisel School of Medicine, Dartmouth College, Hanover, NH USA; 6 Faculty of Health Sciences, Simon Fraser University, Burnaby, BC Canada; 7 Channing Division of Network Medicine, Department of Medicine, Brigham and Women’s Hospital, Boston, MA USA

**Keywords:** Fluoride, Toenail, Biomarker, Reliability, Exposure

## Abstract

**Objective:**

This study aimed to determine the minimum toenail mass needed for reliable fluoride analysis, and to compare fluoride extraction efficiency (%FE) using two protocols.

**Methods:**

Toenail samples (n = 98 from 11 participants) were grouped into masses ranging from 1.0 to 5.0 mg (± 0.5 mg). Fluoride content was analyzed using a hexamethyldisiloxane(HMDS)-facilitated diffusion method and a fluoride-ion selective electrode. Pooled toenail samples (3.0 ± 0.5 mg) from four participants were analyzed using two protocols: protocol-A used 1 mL of HMDS-saturated 3N sulfuric acid/2 mL DI water; and protocol-B used 3 mL/3 mL. Samples were re-analyzed twice to calculate %FE. Minimum mass-threshold was determined by intraclass correlation coefficients (ICC) with 95%-confidence intervals (CI) from linear mixed-effects models and change-point analysis, accounting for intra-participant variability. F-tests compared the variance in %FE between protocols.

**Results:**

Fluoride content ranged from 0.82 to 9.22 µg/g. Excellent reliability (ICC = 0.90, 95%CI:[0.71, 0.95]) was observed in samples ≥ 2.5 mg. A change-point was identified at 2.20 mg(95%CI:[1.70, 2.70 mg]). A less variable %FE was observed with protocol-A (43–58%) compared to protocol-B (38–80%) (F-ratio = 0.23, p = 0.02).

**Conclusion:**

Toenail sample mass affects the reliability of fluoride analysis. The use of a minimum toenail mass of about 2.2–2.5 mg increases the reliability of fluoride analysis at the participant level. Variability in %FE should be considered in future efforts to standardize analytical protocols.

**Supplementary Information:**

The online version contains supplementary material available at 10.1007/s13530-026-00321-y.

## Introduction

Toenails are often used as non-invasive biomarkers in epidemiological research due to their practical advantages, such as simpler collection and storage procedures. Compared to fingernails, they are generally less prone to external contamination [1, 2] and have a slower growth rate, making them well-suited for evaluating long-term exposure to environmental and chemical agents, such as trace-elements and non-metals [2], including fluoride [1].

Fluoride, the negatively charged ion of the element fluorine, is widely present in nature and in the human diet. Due to its well-established role in preventing dental caries [3], fluoride is commonly added to oral healthcare products like toothpastes and mouthwashes and is applied topically in clinical settings. It is also added to drinking water, salt, and milk as a public health initiative in some regions [4]. However, excessive fluoride intake is associated with adverse effects on mineralized and non-mineralized tissues [5–7], and with potential adverse health outcomes, including reduced child IQ [8, 9] and disruptions to thyroid function [10]. These concerns highlight the growing need to better understand and monitor fluoride exposure across different populations.

After ingestion, fluoride is primarily absorbed in the stomach and small intestine, a process influenced by factors such as gastrointestinal pH, nutrient interactions and overall dietary composition [11]. Once absorbed, fluoride is distributed via the bloodstream, with plasma concentrations affected by factors such as bone turnover, renal function and the body’s acid–base balance [12]. Due to its high affinity for mineralized tissue, fluoride is primarily deposited in hard tissues (mainly bone) where it is strongly —though not irreversibly— bound. In contrast, only ~ 1% is deposited in soft tissues. Circulating fluoride is also incorporated into the nail’s matrix, and its content in nails depends on factors that influence the nail’s growth rate and length [13]. Fluoride detection in fingernails and toenails has been reported to reflect exposure over a period of approximately 3–4 months [13, 14]. This has supported the use of nails as a biomarker of longer-term exposure, particularly in association to dental fluorosis [15, 16].

Compared to other biomarkers of fluoride exposure, toenails are considered biomarkers of recent exposure [17]. While contemporary biomarkers, including blood/plasma, saliva, and urine, capture shorter-term fluctuations, they reflect different periods of exposure, with blood/plasma reflecting exposure within the last 3–6 h, saliva over the last few hours (as it follows plasma levels) and urine over the preceding 24 h [18]. Historical biomarkers such as bone and teeth, provide a longer window but require invasive sampling [18]. Compared to hair, also a keratinized matrix, toenails are less prone to external contamination and provide stronger, often more consistent, correlations with fluoride intake [19].

A range of techniques has been employed for fluoride analysis in nails, including neutron activation analysis [20, 21], acid digestion/extraction methods [22, 23], gas chromatography [24], colorimetric spectrophotometry [25], and the most frequently reported, hexamethyldisiloxane (HMDS)-facilitated diffusion method [14, 26]. However, the potential impact of variations in methodologies and protocols for analyzing fluoride in the nail matrix using HMDS-facilitated diffusion techniques requires further research aiming for standardization [27].

The HMDS-facilitated diffusion method is used to release fluoride by acid-induced dissociation of HMDS, an organosilicon compound [28]. In the presence of a strong acid such as sulfuric acid (H_2_SO_4_), HMDS reacts with hydrogen fluoride (HF) generated from the sample or standard, forming the volatile compound trimethylfluorosilane. This compound diffuses into an alkaline trap, where the fluoride ion is released by exchanging with a hydroxyl ion from the trap, and is then measured using an ion-selective electrode. For fingernail specimens, studies have reported fluoride analysis through HMDS-facilitated diffusion using a minimum of 5.0 mg of nail collected from adults [14]. However, to date, no studies have investigated the impact of low toenail sample mass, defined in this study as less than 5.0 mg, on the reliability of fluoride analysis using the HMDS-facilitated diffusion method. There is also limited understanding of whether analytical protocols using the HMDS-facilitated diffusion method introduce variability in fluoride extraction efficiency (%FE) from toenails, which evaluation could advance methodological standardization. Therefore, the objectives of this study were (1) To determine the minimal mass required for reliable fluoride analysis in toenail samples, and (2) to compare the %FE from toenail samples using two different protocols for HMDS-facilitated diffusion.

## Materials and methods

### Study design

A methodological study was conducted to determine the minimum toenail mass required for reliable fluoride analysis and to compare the fluoride extraction efficiency of two different laboratory protocols.

### Sample collection

Toenail specimens with mean pooled mass of 49.5 ± 26.8 mg (range: 22.4–104.6 mg) were collected from a convenience sample of 11 female participants with mean age of 30.7 ± 8.7 years. The participants were residents of the city of Toronto, Canada, and five (45.5%) were between 15 and 38 weeks of pregnancy. Pregnant and non-pregnant participants were included based on previous research indicating no differences in nails’ composition due to pregnancy [29]. All analyses were conducted using the same group of participants in order to eliminate potential variations in exposure and pinpoint differences in methodological procedures. Participants were instructed to remove any toenail polish or varnish and to clip their toenails after a bath or shower. Then, participants placed either big toenail clippings or other toes’ clippings in separate plastic collection bags. All participants signed a written informed consent form prior the study procedures and authorized the use of their samples for study purpose. Toenail specimens were stored and shipped to Indiana University School of Dentistry’s fluoride research laboratory for analysis. Study procedures were approved by the York Research Ethics Board in Toronto, Canada.

### Sample preparation

**Fig. 1:**
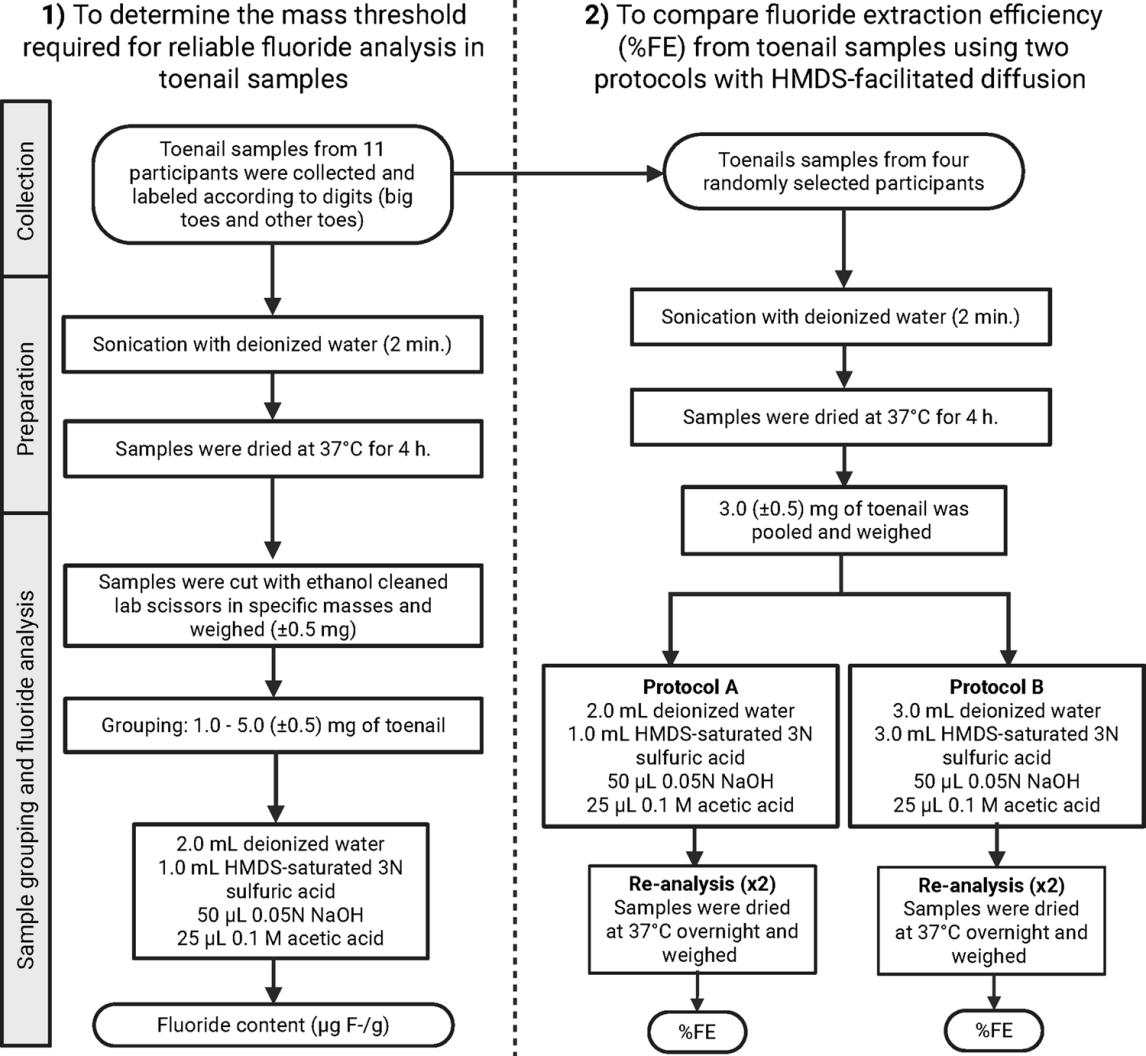
Study procedures for determining mass threshold and fluoride extraction efficiency (%FE) in toenail samples

Pooled toenail specimens were cleaned by sonication in deionized water (diH2O) for 2 min and dried at 37 °C for 4 h (Fig. 1). Toenails from each participant were randomly selected and cut using ethanol-cleaned lab scissors, weighed and grouped into specific masses ranging from 1.0 to 5.0 mg (± 0.5 mg) to create replicate samples (Table 1) for each toe type (big toes and other toes), depending on the availability of specimens’ masses. Only four participants provided toenail clippings from both types of digits.

**Table 1:** Summary of replicate toenail samples and mass characteristics per participant

Participant	Number of replicate samples^a^	Mass (mg) Mean ± SD	Mass (mg) Minimum–Maximum
1	10	3.09 ± 1.43	1.1–4.9
2	4	2.00 ± 0.90	1.1–2.9
3	10	3.18 ± 1.42	1.5–5.5
4	5	3.00 ± 1.86	0.8–5.5
5	10	2.75 ± 1.33	0.9–5.1
6	10	3.10 ± 1.42	1.1–5.1
7	5	3.20 ± 1.62	1.2–5.4
8	20	2.97 ± 1.44	0.8–5.2
9	10	3.12 ± 1.50	1.3–5.5
10	10	2.93 ± 1.46	0.9–4.8
11	4	2.43 ± 1.26	1.2–4.1

^a^The number of replicates was limited by the availability of participants’ toenail specimens

A total of 98 toenail replicate samples were prepared for fluoride analysis. The average number of replicate samples (± SD) analyzed per participant was 9 (± 5) with a range of 4–20. The range of mass of toenail samples was 0.80–5.50 mg (Table 1).

### Determination of fluoride content

Figure 1 summarizes the study procedures. The present study used the procedure described by Martinez-Mier et al. (2011). First, each toenail specimen was placed on the bottom of a disposable 60 × 15 mm Petri dish, and 2.0 mL of diH2Owere added into each dish. After applying petroleum jelly to the inside of each Petri dish lid, 50 μL of 0.05 N sodium hydroxide (NaOH) solution was placed in five equal drops on each dish lid. Each dish was then immediately tightly sealed. After burning a small hole into each lid with a soldering iron, 1.0 mL of HMDS-saturated 3 N sulfuric acid (H_2_SO_4_) was pipetted in each hole and sealed immediately with petroleum jelly. After overnight diffusion at ambient temperature, fluoride was released and trapped in the NaOH. The trap was recovered and buffered with 25 μL of 0.1 M of acetic acid. The recovered solution was adjusted to a final volume of 100 μL with diH2O. The concentration of fluoride was then measured using a fluoride-ion selective electrode (with a limit of detection of 0.019 mg/L) coupled to a pH/ISE meter (Orion™ Fluoride Electrode and Dual Star™ pH-meter, Thermo Scientific, Waltham, MA, USA). A calibration curve using tenfold serial dilutions of Orion™ ISE standard solution traceable to the National Institute of Standards and Technology (ranging from 0.001 to 2.00 µg/mL) was constructed following the same procedure. Millivolt readings from the samples were recorded and the unknown fluoride content from toenail samples were determined using the equation that explained the relationship between the log of the fluoride concentration of the standards and their corresponding millivolt readings (*R*^2^ > 0.99).

### Quality control procedures

Assay precision was evaluated using the daily calibration standards over four days of analyses. The mean intra-assay precision (coefficient of variation [CV] between daily duplicates across all standards) was 5.1%. The inter-assay precision (CV for each standard across the four analytical days) was 8.2%.

### Fluoride extraction efficiency and protocol comparison

Toenails specimens from four randomly selected participants were used to compare fluoride extraction efficiency (%FE) using two protocols for HMDS-facilitated diffusion (Fig. 1). Only nail specimens from other toes (excluding big toe) were used to minimize potential variability in fluoride content by toe type. After sonication with diH2O and drying as described above, six replicate samples weighing approximately 3.0 (± 0.5) mg were pooled from each participant. Then, three replicate samples from each participant were randomly assigned for fluoride analysis using Protocol A and three to Protocol B, yielding 12 replicate samples assigned per protocol. Each replicate sample underwent three sequential analyses (initial analysis followed by two re-analyses), resulting in 36 fluoride measurements per protocol. The two protocols use HMDS-facilitated diffusion but differed in the amount of reagent and water: Protocol A used 1.0 mL of HMDS-saturated 3 N H_2_SO_4_ and 2.0 mL of diH2O (see section on determination of fluoride content), while Protocol B used 3.0 mL of HMDS-saturated 3 N H_2_SO_4_ and 3.0 mL of diH2O. %FE was calculated as the amount of fluoride (mg) extracted in each analysis divided by the sum of fluoride amount (mg) extracted across all analyses, multiplied by 100. The fluoride amount (mg) was determined by multiplying the sample weight (g) by its fluoride content (µg/g) and converting to milligrams (mg). For each analysis, samples were dried at 37 °C overnight and weighed to account for changes in toenail mass following HMDS-saturated acid and water exposure.

### Statistical analyses

Descriptive statistics were used to summarize the fluoride content in toenail samples, including measures of central tendency (median) and dispersion (interquartile range [IQR] and range). To assess variability in fluoride content by toe type (big toes versus other toes), a mixed-effects regression model with inverse-transformed fluoride as the outcome and robust standard errors was fitted, accounting for within-participant replicates. To assess within-subject variability and establish a minimum toenail mass threshold for reliable fluoride measurement, intraclass correlation coefficients (ICCs) were calculated for 0.50 mg mass increments using the variance components of linear mixed-effects models, specifying participant as a random intercept. Interpretation of ICC values followed Koo and Li [30], where values < 0.5 indicate poor, 0.5–0.75 moderate, 0.75–0.9 good, and > 0.90 excellent reliability. Bootstrapped 95% confidence intervals were estimated using 1,000 resamples in the mixed-effects modelling. Change-point analysis with cluster bootstrapping [31] was conducted to detect the mass at which a significant shift in toenail fluoride content occurs as a function of toenail mass, accounting for participant-level variability. The 95% confidence interval for the change-point was estimated using 1000 cluster bootstrap replicates. Normality of the %FE distribution for both protocols was tested using the Shapiro—Wilk test (p > 0.05). The variance in %FE between protocols was compared using an *F*-test for equality of variances. Statistical significance was set at p < 0.05. All analyses were performed in R (version 4.4.2) and Stata (version 19, Stata Corp).

## Results

### Determination of minimal mass required for reliable fluoride analysis in toenails.

The median (IQR) fluoride content across all toenail samples (n = 98) was 2.07 (1.53) µg F^−^/g, ranging from 0.82 to 9.22 µg F^−^/g. When comparing the fluoride content in toenails according to toe type, nails from big toes (n = 25) showed a higher median (IQR) [1.98 (1.50) µg F^−^/g] than those from other toes (n = 25) [1.55 (1.27) µg F^−^/g]; however, this variability by toe type was not statistically significant (*p* = 0.32).

**Fig. 2:**
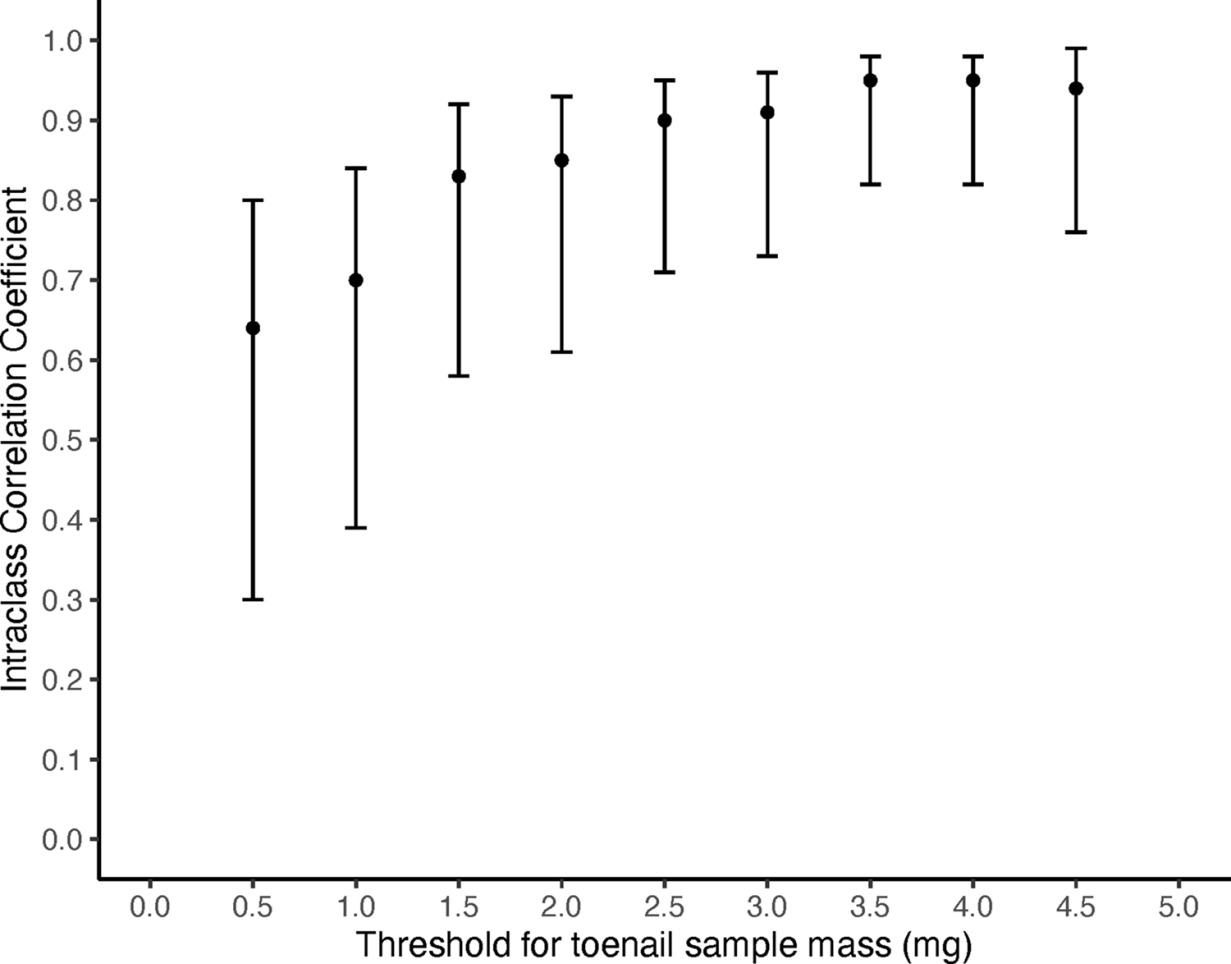
Determination of mass threshold for reliable fluoride analysis in toenails. Intraclass correlation coefficients with 95% confidence intervals for 0.5 mg increments

The distribution of fluoride content in toenail versus replicate sample mass for each participant is presented in S1 File. Mixed effects models were run to determine total and between-subject variance, and calculated intraclass correlation coefficients (ICCs) for 0.5 mg increments of toenail mass. It was observed that with a minimum toenail mass of 2.5 mg, fluoride measurements showed very high to excellent reliability (ICC = 0.90, 95% CI: [0.71, 0.95], Fig. 2). Similarly using a change point analysis fluoride content significantly shifted at a toenail mass of 2.2 mg (95% CI: [1.70, 2.70 mg]), appearing unrelated to mass above that mass.

### Comparison of fluoride extraction efficiency from toenail samples using two different protocols with HMDS-facilitated diffusion.

Table 2 presents the fluoride content and fluoride extraction efficiency (%FE) for each set of analysis or re-testing of the same samples. Median fluoride content and %FE varied across analyses for both protocols, with Protocol A showing a narrower range of %FE values compared to Protocol B. Specifically, the variance of %FE for Protocol A (*var* = 32.0) was significantly lower than the variance of %FE for Protocol B (*var* = 137.7), with *F* = 0.23 and *p* = 0.02.

**Table 2:** Fluoride content and fluoride extraction efficiency (%FE) for each set of re-analysis of toenail samples

	Initial analysis		First re-analysis		Second re-analysis	
	Median (IQR) µg F^−^/g	Range %FE^a^	Median (IQR) µg F^−^/g	Range %FE^a^	Median (IQR) µg F^−^/g	Range %FE^a^
Protocol A (N = 36)	0.76 (0.35)	42.74–58.32	0.30 (0.10)	13.10–28.80	0.36 (0.18)	21.42–33.46
Protocol B (N = 36)	0.60 (0.31)	38.12–82.65	0.29 (0.18)	18.99–35.18	0.15 (0.15)	9.64–28.07

^a^%FE: Fluoride extraction efficiency was calculated as the amount of fluoride (mg) extracted in each analysis divided by the sum of fluoride amount (mg) extracted across all analyses, multiplied by 100. The fluoride amount was determined by multiplying the sample weight (g) by its fluoride content (µg/g) and converting it to mg

## Discussion

The findings from the present study indicate that toenail sample mass affects the reliability of fluoride analysis when using the HMDS-facilitated diffusion technique. Additionally, certain modifications to the HMDS-facilitated diffusion method, such as increasing the amount of HMDS-saturated sulfuric acid and deionized water, were found to affect variability in fluoride extraction from toenail samples.

To our knowledge, this is the first study to examine the impact of low toenail sample mass (< 5.0 mg) on the reliability of fluoride analysis using the HMDS-facilitated diffusion method. Whitford [26] reported that fingernail clippings in the 10–20 mg range typically contain sufficient fluoride for accurate analysis with this method. However, in epidemiological studies, only much lower toenail masses may be available and the reliability of using the HMDS-facilitated diffusion technique with low mass toenails was unknown. In the present study, it was found that a minimum toenail mass of 2.5 mg can yield good to excellent reliability (95% CI for ICC: [0.71, 0.95]) in estimating fluoride content at the participant level when analyzing toenail samples from adults. In contrast, low mass toenails (i.e., below 2.5 mg) were less reliable. Evidence from studies using other analytical techniques has shown that toenail sample mass can affect the intra-individual stability of elemental concentrations, including various metals and metalloids, in cohort studies [32]. This is particularly relevant in epidemiological research, where the quantity of collected biological material is often limited and must support multiple environmental exposure assessments. Therefore, standardizing sample collection and analytical protocols is essential for future research.

Previous studies have suggested that smaller mass nail samples may not contain sufficient fluoride to be reliably detected, particularly considering the ion-selective electrode’s limit of detection [26]. In the present study, smaller toenail samples showed reduced reliability of fluoride measurement at the participant level. One plausible explanation for this observation is the structural composition of nails, which are predominately formed by a dense keratinized network that may function as a porous medium facilitating pathways for acid penetration [33], thereby potentially increasing the variability in acid diffusion for smaller sample masses with the HMDS facilitated extraction process. An additional finding was that fluoride may not be fully extracted from the specimen matrix in a single analysis, as indicated by variable extraction efficiency in repeated tests. The nail's structural composition as a dense keratinized network and acting as a porous medium may impede complete acid penetration and fluoride release in a single extraction process. Lower extraction efficiencies suggest that the fluoride content reported using this method may underestimate the total fluoride content bound within the nail matrix. From an epidemiological perspective, incomplete extraction may be acceptable provided it remains consistent across samples, thereby preserving the relative ranking of participants' exposures. However, higher variability in extraction efficiency can lead to non-differential exposure misclassification in studies using toenail fluoride for associations with health outcomes. Future studies should evaluate extraction efficiency across a range of toenail sample masses to better understand its contribution to measurement variability and improve standardization.

The HMDS-facilitated diffusion is a gold standard technique [27] for liberating fluoride from various non-biological and biological samples, including nail clippings [26]. In the present study it was observed that increasing the amount of HMDS-saturated acid and water in the solution, increased the variability in fluoride extraction across multiple set of analysis of pooled toenail samples. This finding could be explained by assuming that a larger volume of HMDS-acid saturated and water in the solution provides a higher amount of reactants. This in turn translates into higher amounts of HF in the solution that do not reach the NaOH trap, increasing the chance of varied extraction from the nail matrix. The use of different volumes for reactants could impact overall fluoride content and therefore use of a standard protocol should be used when comparing toenail fluoride content across studies. Studies on the mechanistic aspects are needed to understand the impact of modifying the liquid phase for this technique when analyzing toenail samples.

Toenails remain a less invasive biomarker compared to other biological media and are easier to collect, offering practical advantages in epidemiological research [1]. Additionally, toenails reflect longer exposure periods, approximately 3–4 months, which can be useful for studies examining exposure over time, such as the course of pregnancy. Another advantage is that toenails integrate fluoride exposure from multiple sources (e.g., drinking water, diet, dental care products), potentially serving as a biomarker of sub-chronic exposure, a feature that is an ongoing question when investigating associations with human health outcomes. Future research should examine the impact of demographic factors, such as biological sex and age to study whether these factors can introduce potential changes in fluoride accumulation in toenails and assess biomarker reliability across varied populations.

This study had some limitations. Previous studies have suggested that nail growth rate and length should be assessed in study participants, as these factors may influence fluoride content estimates [13]. However, investigating the impact of growth rate was beyond the scope of the present study. In addition, this study did not collect information to assess fluoride intake or exposure data from the participants, which limits the comparison of fluoride content in toenails reported in other settings, including those with community water fluoridation. Additionally, while the study included 98 analytical replicates, these were derived from 11 participants, which may over-or underestimate ICC. Furthermore, according to ISO 5725-1 metrology guidelines [34], while our ICC analyses aimed at establishing the reliability (i.e., repeatability and reproducibility) of the measurements, determining overall analytical accuracy requires establishing trueness (validity). Because a certified reference material for fluoride in a keratinized tissue matrix was unavailable, trueness could not be established. Despite these limitations, this study has the advantage of including the use of a gold standard method [27] for fluoride analysis for the determination of sample mass threshold and the comparison of extraction efficiency for toenails samples.

## Conclusions

In summary, toenail sample mass affects the reliability of fluoride analysis using the HMDS-facilitated diffusion method, with lower masses showing reduced consistency in fluoride estimates. A minimum mass of about 2.2–2.5 mg was found to provide good to excellent reliability, highlighting the importance of defining minimum sample requirements. Additionally, modifications to the HMDS protocol may affect fluoride extraction variability, emphasizing the need for standardized analytical procedures, especially if comparing fluoride content across studies. Although toenails offer practical advantages as non-invasive, long-term biomarkers of fluoride exposure, future research should address protocol variability to improve reliability in epidemiological applications.

## Supplementary Information

Below is the link to the electronic supplementary material.

Supplementary file1 (DOCX 71 kb)

## Data Availability

The data that support the findings of this study are available from the corresponding author, GTC, upon request.
