# Supplementary material for: Impact of sample mass on the reliability of toenail fluoride for exposure assessment
Source: Toxicol Environ Health Sci. 2026 Apr 27;18(3):431–8. doi: 10.1007/s13530-026-00321-y (PMC13473185; doi:10.1007/s13530-026-00321-y)
Supplement: Supplementary file 1 [file 13530_2026_321_MOESM1_ESM.docx]

**Figure.** Participant-level distribution between toenail sample mass and fluoride content.
